# Country-specific food culture and scientific knowledge transfer events – Do they influence the purchasing behaviour of seafood products?

**DOI:** 10.1016/j.aquaculture.2022.738590

**Published:** 2022-11-15

**Authors:** J. Petereit, C. Hoerterer, G. Krause

**Affiliations:** a Alfred Wegener Institute Helmholtz Centre for Polar and Marine Research, Am Handelshafen 12, 27570 Bremerhaven, Germany; b Institute for Advanced Sustainability Studies (IASS), Potsdam, Germany

**Keywords:** Sustainability, Consumer analysis, Europe, Seafood, Perception

## Abstract

A positive perception of aquaculture products is essential to boost production by using more sustainable and eco-friendly solutions. However, consumer perception and resulting purchasing decisions remain poorly understood. In most European countries, the consumer perception tends to be rather negative, which is reinforced by knowledge gaps and misleading information from the media. This is believed to have the greatest impact on the current low consumption rate of farmed fish across Europe. Previous research has suggested that consumers may often be reluctant to change their seafood purchasing behaviour despite having a solid scientific understanding of aquaculture products and their mode of production. In this study, we investigated the extent to which country-specific contexts and degree of scientific knowledge contribute to the purchasing behaviour of consumers across Europe. To this end, interactive poster surveys and semi-structured interviews were conducted at eight different knowledge transfer events (KTEs) across three countries, targeting 383 participants. The application of a yet underutilized method, an interactive poster survey, underscored the need to use new approaches to tackle consumer behaviour.

Our results indicate that increased scientific knowledge does not lead to changes in purchasing behaviour per se. Perceptions and purchasing habits are very contextual and vary from culture to culture. This points to the highly interlinked nature of country-specific marine food culture that ranges between individual awareness, scientific knowledge, and socio-cultural contexts, all of which renders in resulting individual purchasing decisions. Our results suggest focusing more on the sustainability of a product and emphasising the ongoing transition towards a circular economy approach in the aquaculture sector may be a promising pathway to foster more sustainability-driven purchasing decisions in the seafood sector. Our findings also question whether trying to educate the public about more sustainable purchasing criteria is really the key to foster more sustainable consumption patterns or whether we are working from misleading assumptions that lead to wrong approaches. In conclusion, a lack of clear and easily accessible information appears to be the main barrier to social acceptance of sustainable aquaculture products in Europe.

## Highlights

• Focusing more on the sustainability of the product and emphasising the need for a circular economy approach could foster more sustainability driven purchasing decisions.
• Our study showed for the first time that increased awareness does not necessarily translate into increased attention in the supermarket when consumers purchase seafood.
• The circular economy approach fosters more sustainable and transparent products and provides biodiversity and ecosystem services, leading to better consumer perception.

## 1 Introduction

Roughly 60% of the world's marine fish stocks are fully exploited, and 33% are overfished all of which call for transformation in the seafood sector (FAO, 2018, López-Mas et al., 2021). This is especially timely in the European Union, where consumers use three times more seafood than is produced, making Europe the largest importer of seafood in the world (Krešić et al., 2020).

Under this umbrella, marine aquaculture is believed to boost the highest transformative potential towards more sustainable pathways of future food security (Altintzoglou et al., 2020; Eurobarometer, 2018; Krešić et al., 2020; Hackenesch et al., 2016). One approach to promote sustainable aquaculture growth is the concept of eco-intensification, i.e., production sufficient to meet the needs of the human population while respecting environmental needs and promoting health benefits (Aubin et al., 2019; O'Donncha and Grant, 2019; Pieniak et al., 2010). Eco-intensification is a challenge that requires the integration of scientific and technical innovations as well as addressing social considerations to promote the implementation of the principles of circular economy in aquaculture (Aubin et al., 2019; Føre et al., 2018). Seafood has positive health attributes, such as reduced cardiovascular disease risk or better neural development during childhood (Boase et al., 2019; Menozzi et al., 2020; Willett et al., 2019). Thus, especially the health benefits seem to have a high influence on the purchasing behaviour of the consumer (Hoque and Alam, 2020; Pulcini et al., 2020; Wongprawmas et al., 2022).

However, the general perception of aquaculture products in most European countries tends to be negative, e.g., due to concerns about animal welfare, potential climate impacts on aquaculture production, as well as general sustainability characteristics (Alexander et al., 2016; Claret et al., 2016; Feucht et al., 2018; Hoerterer et al., 2022a; Wongprawmas et al., 2022). This negative perception is believed to have the greatest impact on the low consumption rates of farmed fish in Europe and can be mainly linked to false and misleading information in the media (Govaerts, 2021; Heide and Olsen, 2017; Krešić et al., 2020). This misrepresentation of aquaculture and the resulting knowledge gaps, especially in Europe, leads to a prevailing overall negative perception of farmed seafood (Froehlich et al., 2018; Hoque and Alam, 2020; Thomas et al., 2018) and leads to low consumption rates of aquaculture products in Europe (Banovic et al., 2019; Boase et al., 2019; Gaviglio, 2009; Menozzi et al., 2020; Schlag, 2013). The literature suggests that the key drivers of seafood consumption are demographics, knowledge, and personal background (traditions, culture, etc.) (Boase et al., 2019; Verbeke et al., 2007; Vinayak and Arora, 2018). These need to be considered when promoting sustainable eco-intensification of aquaculture production, given the social boundaries closely linked to the labour market and public health (Gerten et al., 2020; Raworth, 2017). Thus, the only thing that can be actively changed in a society is to increase awareness and knowledge, which is why it is crucial to understand the level of knowledge on the individual consumer level (Boase et al., 2019; Krešić et al., 2020; Wongprawmas et al., 2022).

In light that consumers often have limited knowledge about aquaculture in general, it is assumed that positive attitudes, shaped by improved scientific knowledge towards seafood, can increase its consumption and thus influence the overall sustainability of this sector (Krešić et al., 2020). Therefore, the increase in consumption and awareness is related to companies placing more importance on and promoting of these factors. However, despite increasing awareness and campaigns aimed at changing consumer habits towards buying sustainable aquaculture products, overall consumer choices of seafood are still poorly understood and highly complex (Almeida et al., 2015; Hughes and Black, 2016; Moschitz et al., 2021; Solgaard and Yang, 2011). Indeed, to find an influential market strategy, it is crucial to understand consumer preferences, perceptions and choices (Menozzi et al., 2020; Hoque and Alam, 2020).

Under this premise, the EU Horizon 2020 project GAIN (Green aquaculture intensification in Europe funding N° 773,330) commenced in 2018. This project has a strong focus on investigating the potential of ecological intensification of European aquaculture. Key elements of this project are feed improvements, circular economy, precision aquaculture, policy, and markets. Drawing on a transdisciplinary approach, research efforts are focused on integrating scientific and technical innovations, new policy and economic instruments, and reducing social constraints, i.e., identifying and addressing the prevalence of negative perceptions of aquaculture (https://www.gain2020.com).

Within the framework of the GAIN project, this study investigated:

Research question (RQ 1): What role do cultural preferences of individual countries play in consumer choices?

RQ 2: Does more scientific knowledge about seafood products lead to increased consumer awareness and thus hosts the potential to positively change purchasing behaviour towards sustainable aquaculture products? More specifically, does improved scientific knowledge transfer in different European Union countries has the same effect on the purchase of aquaculture seafood products?

RQ 3: How can we positively nudge the consumers perception and acceptance towards more sustainable aquaculture seafood products?

To address these somewhat intangible research questions and especially the role of scientific information as baseline for individual decision-making therein, a novel mixed-method approach is required. This is elaborated on more detail in the next section.

## 2 Material and methods

This study is the first to look specifically at the role of scientific knowledge rather than general consumer awareness at large. To this end, an interactive poster study approach was selected in order to better exchange with participants and understand their motivations within a rather informal setting. Such interactive poster studies are still rarely used, and more investigations about them are needed to draw better conclusions about their practicability. As such, this is the first study of its kind and is primarily intended to uncover initial trends rather than statistically relevant data, which would be needed in subsequent studies to fully validate these primarily findings obtained here.

Based on an extensive literature search, we identified several criteria that have the biggest influence on the individual purchase decision: price, health benefits, importance of certification, animal welfare, and product processing (Cantillo et al., 2021; Krešić et al., 2020; Menozzi et al., 2020; Pulcini et al., 2020). We decided to focus on these central five criteria in order to make the posters as simple and accessible as possible vis á vis to obtain first insight into the differences concerning the magnitude of available scientific knowledge and its respective influence on individual decision-making. Employing a mixed-method approach (Danermark et al., 2019; Kelle, 2014; Levitt et al., 2018), the selected purchase criteria were quantitatively assessed via an interactive poster survey, followed by a series of semi-structured interviews for descriptive refinement and validation (Dale and Kline, 2017; Esterberg, 2002; Mabrouk et al., 2018; Rowe and Ilic, 2009). This makes this approach particularly effective (Diebold et al., 2017; Michalsky et al., 2018). All methods were pre-tested, and the outcomes of the first surveys were further refined.

### 2.1 Poster surveys

In order to clarify the specific national context of consumers, we conducted a series of interactive poster surveys to validate findings from collated secondary data sources (FAO country statistics and Eurobarometer). This method is highly suitable if unbiased data is needed on a specific topic (Pratt et al., 2000; Salzl et al., 2008; Hilton, 2015). However, a prerequisite for the application is to allow enough space for responses (which we ensured by the subsequently conducted semi-structured interviews) and not to present too many details (Altintzoglou et al., 2018). Furthermore, the format must be clear from the onset and must include the thematic focus of interest. In our case, the focus was 1) to gain an understanding of consumption preferences of aquaculture products and 2) on the importance of individual viewpoints on eco-intensification measures concerning purchase criteria with regard to different scientific knowledge degrees. In this way, it is possible to estimate how much scientific knowledge, expectations, and assumptions participants have about aquaculture at the individual level as a first approximation. Thereby, we follow Esterberg's call (2002) for knowledge exchange as a relationship between two individuals who come together “to try to create meaning about a particular topic,” thereby drawing on a range of different social conventions and cultural knowledge. In this regard, an interactive poster survey is more open, easier accessible and conversational than a structured interview or questionnaire.

#### 2.1.1 Pre-test

The first draft of the poster survey was pre-tested (Pratt et al., 2000; Altintzoglou et al., 2018; Ikart, 2019) at a non-coastal (inland) research institution (Germany) to get a better idea of the perceptions of laypeople that have no professional relationship with aquaculture. Thus, a limited degree of scientific knowledge of aquaculture can be assumed. This pre-test revealed that it is essential to add the option “not eating seafood at all” to the Poster inquiry, as well as to provide room to enquire about other marine species. The pre-test also revealed the problem of language and understanding of the questions, which was modified towards more simple language use in the poster surveys.

#### 2.1.2 Poster survey at knowledge transfer events

Interactive posters are an effective way to engage, communicate, and share information with attendees of all types (Dale and Kline, 2017; Michalsky et al., 2018). In this study, we used this type of poster surveys at different scientific venues as a validation method, which has been shown to be an effective tool when combined with semi-structured interviews (Hulland et al., 2018). Surprisingly, very few studies explore an interactive approach, despite that posters are standard tools at scientific conferences (Diebold et al., 2017; Mabrouk and Schelble, 2018). In addition, previous attempts to motivate scientists to conduct online surveys at conferences are highly challenging (see Hoerterer et al., 2022b). This can be related to the observation that people are rather unwilling to undergo an online survey whilst being more motivated to look at posters and indicate their preferences briefly in an easily accessible manner.

The poster survey aimed to quantitatively identify the prevalent purchase criteria and species preferences (Annex 1). The presented species ranged from marine Atlantic salmon (*Salmo salar*) and sea bream (*Sparus aurata*) to freshwater rainbow trout (*Oncorhynchus mykiss*) since these are the most commonly cultured finfish in terms of value and production volume in Europe (EUMOFA, 2019a; FAO, 2018). In the EU-GAIN project, these three species were the central candidates for circular economy approaches in aquaculture (Hoerterer et al., 2022a; Petereit et al., 2022 in preparation).

However, for the poster survey in Spain, trout was exchanged with the European seabass (*Dicentrarchus labrax*) since this species is much more common in southern Europe and was a further central species for the feed experiments in GAIN (Hoerterer et al., 2022a; Muñoz-Lechuga et al., 2018; Petereit et al., 2022 in manus.).

To clarify the specific national context of consumers, the poster surveys were conducted in different countries employing predetermined questions (PDQ's) as well as one open-ended question (OEQ). The data were collected at eight different knowledge transfer events that were conducted under the umbrella of GAIN outreach activities. These took place between October 2019 and February 2020 in Germany (*n* = 5), Belgium (*n* = 1), Spain (n = 1) and Poland (n = 1) (Table 1).

**Table 1:** Location, estimated total number of participants at the different knowledge transfer events (KTE), no. of participants that engaged with the poster, categorization of participants and respective level of scientific knowledge of the eight KTE.

Country	Total participants	Poster participants	Category	Level of scientific knowledge
Germany	250	113	Young marine scientists	Low
Belgium	Unknown	15	Administrative staff of the EU	Low
Germany	50	26	Marine scientists and technicians	Medium
Spain	150	63	Marine scientists and administrative staff	Medium
Germany	50	35	Seafood specialists	High
Germany	50	27	Fisheries scientists	High
Germany	2700	52	Aquaculture scientists	High
Poland	250	52	Carp aquaculture specialists	High

In total, more than 400 participants took part in the two-page poster survey. The participants consisted of scientists with or without an aquaculture background, aquaculture operators, students, and laypeople across different age groups and with varying levels of scientific knowledge about seafood products. For the latter, detailed online surveys were conducted, and findings were collated and analysed in Hoerterer et al. (2022b). Of these, 383 filled in the more detailed questions on seafood purchasing criteria, next to the questions on species preferences. From these 383 participants we randomly picked some and conducted short semi-structured interview to validate their choices stated in the poster survey (See 2.2). In order to provide a “low-barrier-to-entry” situation, all posters were placed in a public spot where people were able to pass by frequently in a relatively informal manner. This methodological approach has already been tested in various studies and found to be useful in practice (Dale and Kline, 2017; Mabrouk et al., 2018; Michalsky et al., 2018). The results were validated with findings from secondary data sources (Eurobarometer, 2018; FAO, 2020). All responses were self-administered by the participants (i.e., participants completed the survey themselves without interference from the researchers). The subsequent semi-structured interviews took place after the participant finished his/her preference indication on the poster survey. Therefore, the resulting dataset contained fully anonymous and non-identifiable records.

### 2.2 Semi-structured interviews

To validate the poster survey results and obtain more in-depth knowledge, around 40 semi-structured interviews were conducted alongside the poster at the various knowledge transfer events. Semi-structured interviews are a commonly used method to achieve a deeper understanding of human experiences (Bearman, 2019; Dale and Kline, 2017). These semi-structured interviews helped interpret the selections made on the poster, clarified the results, and, by definition, took place in an informal tone and were used to gain more insight into the participant's disclosures (Longhurst, 2004). Recording of the interviews was not conducted as the central purpose of these was to gain more insight into the purchase decision on an individual level and to keep the validation process as unintrusive as possible. In these interviews, participants were asked to justify and explain their decisions in more detail. These were conducted randomly with participants at scientific conferences and similar knowledge transfer events, at which they indicated their consumer preferences via the poster survey. Despite the limitations in regard to representativeness, these provide good insight into the decision-making process of the individual consumer and the role scientific knowledge plays therein.

### 2.3 Description of scientific knowledge transfer events, their focus, and participants' characteristics

We conducted the interactive poster surveys and semi-structured interviews at eight different scientific knowledge transfer events (KTE), addressing respective target groups and various degrees of participation rates (Table 1). In order to categorize collected data by the level of scientific knowledge, we classified the participants of the respective KTE into low, medium, and high knowledge groups after the semi-structured interviews during the survey. This was done out of the recognition that the different KTEs were targeting different audiences with varying degrees of scientific knowledge on aquaculture. These different levels of scientific knowledge surfaced during the poster survey and subsequent semi-structured interviews that allowed categorization of their knowledge base. For instance, the group of “low” scientific knowledge consists mainly of younger participants (e.g., mostly Generation Z and a few Generation Y's) with only a very marginal background in the topic of aquaculture research. This group mainly got their aquaculture knowledge from the media and displayed in general very negative and fixed mindsets. The second “low scientific knowledge” category consisted of educated laypeople that were located inland in a major city that showed an overall low knowledge about different practices and sustainability of aquaculture. In contrast, the “medium” knowledge groups consist of people who stated to have some degree of exposure and knowledge of aquaculture. This category consists mainly of scientists that are not directly involved in aquaculture research but in other marine biology studies (such as sea shelf ecology, fisheries, or general biology studies). The last category with “high” scientific knowledge were experts with a strong degree of scientific knowledge in aquaculture production or related fields and/or were professionally involved in the seafood production sector.

The latter category was the easiest to categorize because the KTE were very specific aquaculture conferences, in which only people within the aquaculture research field were present. The semi-structured interviews revealed here that all participants had an excellent background in scientific knowledge of aquaculture and were e.g. aware of different rearing systems, sustainability issues, and other factors influencing the industry.

For most KTE, we were able to estimate the number of participants at the conference by either asking the organizer or our own rough estimations.

## 3 Results and discussion

Analysing the consumer decision-making process is rather complex as many different contextual drivers act on the individual seafood preference that varies over time. However, in order to shed some light on the impact of scientific knowledge in relation to purchasing behaviours whilst acknowledging country-specific differences, we first present and discuss the findings on a country level (research question 1), followed by an assessment of the influence of respective scientific knowledge levels (research question 2), and closing with an individual-level analysis (research question 3). Rooted in the findings of this multi-level and multi-dimensional analysis, recommendations for future research needs and the prospects of seafood preferences are outlined.

### 3.1 Country-specific differences

To gain insights on the question of whether there are potential country-specific preferences in seafood consumption, the poster surveys were conducted at different knowledge transfer events (KTE) per country (Research question 1). These ranged from Spain as a country with one of the highest per capita consumptions with over 45 Kg live weight/ capita/ year, to Belgium, Germany, and Poland as European countries with decreasingly lower per capita consumption rates (13,5–15 Kg/capita/year) (EUMOFA, 2019b). Participants from six out of eight KTE were explicitly conducted in the specific countries mentioned above. The other two KTE were conferences that had mixed international participation and thus were not assigned to a single country (KTEs 2 and 7).

In the following subsections, the results of the various country-specific aspects of seafood consumption preferences are examined in greater detail. The pre-tests showed that especially the species and food processing could be well mapped between the countries. Therefore, we focus particularly on these aspects, as both species preference and processing were cited as the most important determinants of the purchase decision in the literature and were reinforced in our semi-structured interviews (Cantillo et al., 2021; Krešić et al., 2020; Pulcini et al., 2020.

#### 3.1.1 Finfish species preference per country

All participants (*n* = 383) from the various KTE had a clear preference for salmon (31%), closely followed by sea bream (19%) (Table 2). This is consistent with the results of the Eurobarometer survey, which found that 35% of consumers prefer salmon over other species (EUMOFA, 2019a). One-fifth of participants indicated that they prefer other species than the three main given species and therefore provide information about differences across nationalities (Table 2). In the subsequent semi-structured interviews, the latter participants stated that they do not eat seafood due to vegetarian or vegan dietary preferences*.* On average, 33% stated never to buy fresh seafood products in our study. *These findings are consistent with recent studies that indicate that veganism and vegetarian lifestyles have been growing in recent years (**Saari et al.,* 2021*). This vegan/vegetarian movement is often a very individual decision and, more often than not, strongly linked to peer group pressure. However, the understanding of why specific food lifestyles are chosen is not yet well understood. For instance, vegan and vegetarian lifestyles are also known to reduce the incidence of heart disease and diabetes, leading to better health outcomes (**Menozzi et al.,* 2020*;**Pieniak et al.,* 2010*). In addition to personal preferences, animal welfare and sustainability play a crucial role for the decision to go vegan /vegetarian. Many consumers are, due to various reasons, subject to the prejudice that seafood, in general has a negative impact on the ecosystem, as well as on labour conditions (**Govaerts,* 2021*). These prejudices can be linked to the reinforcement of false media image, that more often than not, remain in the prevailing negative narrative of harmful aquaculture systems, thus ignoring contemporary efforts and achievements made towards sustainable aquaculture production systems. In conclusion, why the vegan lifestyle is increasing and what repercussions this has on the consumption of aquaculture products (e.g. seaweed) is a very challenging issue and needs further investigation.*

**Table 2:** Species preference according to the different nationalities.

Overall Participation	Germany	Spain	Poland	International⁎	Total
	181	117	54	83	435
Salmon	37%	17%	28%	42%	31%
Sea bream	20%	12%	2%	37%	19%
Trout	14%	1%	26%	17%	13%
Seabass	1%	20%	4%	0%	6%
Other	8%	50%	30%	0%	21%
No Seafood	19%	0%	11%	4%	10%

⁎ International includes the participants from knowledge transfer event 2 (*n* = 15) and 7 (*n* = 52).

In comparing the different nationalities, only 8% of German participants stated eating species other than the three listed on the poster survey. That said, up to 68% of all species consumed by German participants can be split between salmon (37%), sea bream (20%) and trout (14%) (Table 2). However, interestingly roughly 19% of German participants reported never eating seafood, which is in stark contrast to Spain, where none indicated such a (non-)preference. This reinforces previous studies, showing that German consumers are known to be attracted to the fish they are most familiar with (Koch et al., 2019). In addition, with the exception of salmon, herring and to some extent, seabream, there is very limited advertising in Germany to guide consumers to alternative seafood species. In contrast, Spain has the most diverse species consumption composition, with about 50% indicating to consume none of the three most frequently mentioned seafood species in Europe as a whole, but rather consume a diverse set of other marine species. Contrasting this diverse seafood preference picture in Spain, 30% of seafood consumed in Poland is not covered by the top three preferred marine species in Europe but refers to other, mainly fresh water species, such as perch (31%) and carp (38%).

Country-specific differences between the selected EU countries can be further identified by focusing on the distribution of “other” seafood consumed. Participants were asked to indicate seafood species other than the species listed on the poster. For example, in the semi-structured interviews, Spanish participants indicated that they strongly preferred local (marine) species as long as they were caught nearby and thus indicated a strong recognition and motivation to support their local fisheries and working waterfront communities.

#### 3.1.2 Processing preferences per country

There are considerable differences between the respective EU countries in terms of the degree of consumer preference for processed seafood. Overall, most respondents (65%) indicated to prefer fresh seafood over frozen (17%) and processed products (6%) (Table 3). Our survey results mirror the findings of the Eurobarometer survey (2018) that found similar trends across the EU, where fresh products (37%) are preferred over frozen products (25%). However, the most distinct distribution in preference for processed seafood was found in Poland. Here, 56% of respondents prefer fresh fish over frozen (21%) and processed products (12%), and 12% said they never eat seafood (Table 3). Regarding the consumption of frozen products, all survey participants from Germany, Poland and Spain indicated that they buy such products at least occasionally. However, 76% of all Spanish responders stated to prefer fresh fish over frozen (22%) or processed (2%) fish, and none declared not eat seafood at all. This finding is also reflected in the 2018 Eurobarometer data, which shows that consumers from Spain, followed by Greece, are most likely to buy fresh seafood products (EUMOFA, 2018).

**Table 3:** Processing preferences divided for the different countries.

Overall Participation	Germany	Spain	Poland	International⁎	Total
	201	63	52	65	381
Fresh	58%	76%	56%	80%	65%
Frozen	18%	22%	21%	8%	17%
Processed	5%	2%	12%	9%	6%
No seafood consumption	18%	0%	12%	3%	12%

⁎ International includes the participants from knowledge transfer event 2 (*n* = 15) and from 7 (*n* = 52).

These country-specific differences mirror the respective social-cultural settings in which seafood consumption has been traditionally placed. For example, Spain has a long fishing tradition, which explains the prevalence and acceptance of a wide variety of fish species and the high percentage of consumers who prefer fresh fish. Indeed, traditionally Spanish consumers go to the market and buy fish offered by local fishermen instead of choosing the cheapest processed fish product in the supermarket (Jacobs et al., 2015).

Our surveys reinforce this, whereas 80% of international and Spanish participants indicated that they prefer fresh fish over frozen or processed products. However, these results must be taken with some level of caution due to the low overall number of participation and the fact that all Spanish participants were “economically highly affluent”. Thus, this observed trend in our surveys may not reflect the entire country of Spain. However, it nonetheless can still be reconciled with the results of the semi-structured interviews, where respondents indicated that they strongly support their local fisheries by purchasing seafood products directly from the (local) market.

### 3.2 Differences in the level of scientific knowledge

In the following, we will explore the question of how the degree of available scientific knowledge about seafood products influences consumers purchasing behaviour (Research question 2). Generally, it is assumed that a rise in scientific knowledge about a given product leads to more sustainable purchasing decisions due to the increasing awareness of the problem (Almeida et al., 2015). However, this assumption is difficult to validate due to biased perceptions of the consumers surveyed and categorization of the scientific knowledge itself. Indeed, personal perspective surveys always inherit a factor for error, especially on contested and highly normative topics such as individual food preferences.

#### 3.2.1 Finfish species preference in relation to the degree of scientific knowledge

Drawing on the knowledge transfer events and especially focusing on KTE 1, a conference for young marine scientists with relatively little prior scientific knowledge about seafood production, it is noteworthy that more than one-third emphasized in the semi-structured interviews not eating any seafood or fish at all (37%). Moreover, this was reinforced by the poster survey during this KTE that the participants largely did not indicate any seafood species other than the three suggested on the poster. Those who did indicate consuming seafood stated to prefer salmon (30%), followed by sea bream (18%) and trout (15%). In contrast, the results for the KTE 7, where all survey respondents inherited a high level of scientific knowledge about aquaculture and its products, show only a marginal 5% proportion of non-seafood consumers. Almost half of all participants of the latter KTE stated salmon as their favourite fish species (47%). Sea bream came in second at 32%, followed by trout at 16%.

Most of the participants at the KTE 1 indicated in both survey formats (poster survey as well as subsequent semi-structured interviews) to be highly concerned about healthy lifestyles and the importance of healthy foods in general, which resulted in their decision towards a vegan lifestyle. Indeed, healthy foods are becoming more and more trending, especially in the western world, where healthy diets receive increasing attention among groups with a higher degree of scientific knowledge (Saari et al., 2021; Tomić et al., 2017). Long-chain polyunsaturated fatty acids, like Omega-3, are highly present in fish and are very beneficial for human health. Hence, these are advocated by governments worldwide (Tomić et al., 2017; Turchini et al., 2011; Verbeke et al., 2007). Despite these widely known facts, most of the young marine scientists participating at the KTE 1 indicated they do not eat seafood despite knowing about its health benefits. These results support the hypothesis that scientific knowledge about seafood products is not necessarily per se a driver of purchases. Rather, purchasing seems to be related to situative individual decisions driven by cultural practices, peer-group pressure, and world views.

#### 3.2.2 The role of certification in relation to the degree of scientific knowledge

In most cases, participants' response to whether certification is a purchase criterion was above 50% at all KTE. This indicates that certification as a purchase criterion is important, but not as strongly decisive as commonly believed (Asche and Bronnmann, 2017). However, our results show that at all events where participants inherited a high scientific knowledge, an average of 64% indicated that certification is essential for making a purchasing decision. In contrast, 53% of those with low scientific knowledge and 60% of those with medium scientific knowledge stated that certification is an important aspect of their decision-making. These results show some degree of correlation between the degree of available scientific knowledge and the importance of product certification as a purchase criterion. However, our questionnaire did not consider the differentiation between aquaculture products or wild-capture fish. In our pre-test survey we encountered that many consumers appeared to ignore the type of product (wild-capture or aquaculture), but solely placed their decision making upon the certificates, as they assumed that this indicated a higher degree of sustainability. This links Alfnes et al. (2018) findings that consumers demand traceability. Overall, these results emphasize the connection between higher scientific knowledge and an increase in awareness of the product's sustainability. This can be understood as a central incentive to obtain certification from a producer's point of view and shows the trend of rethinking consumer purchase choices. Indeed, the semi-structured interviews reinforced that most participants were well aware of the MSC logo, while only a few stated to ever have paid attention to its aquaculture counterpart, the ASC logo.

### 3.3 Other purchasing criteria

The remaining criteria listed in our surveys did not show any difference in terms of country-specific or scientific knowledge specific purchasing criteria. For instance, no differences were found between knowledge events regarding animal welfare purchasing criteria (89% overall) and health benefits (64% overall). It is noteworthy that animal welfare was considered very important in all events and across all knowledge levels, nationalities and age groups. Next, all survey results exposed the decisive role of price and origin as purchase preferences. These cannot be related to having distinct country-specific or scientific knowledge availability dimension. Therefore, the following central findings for both criteria are collated.

#### 3.3.1 Price preferences

The price of fish as a purchase criterion is closely linked to the socio-economic positioning of the respective consumer. This affects how much attention is paid to the price of a seafood product. As our surveys exposed, the majority (73%) stated that the price range influenced their purchasing behaviour, and only 29% said they were not concerned about price. The latter is reinforced by earlier findings that show consumers mainly eat fish for its health, nutritional properties, and taste but care less for its origin and more for the price in general (Brunsø et al., 2009; Vanhonacker et al., 2013).

Our surveys revealed that both younger people and noteworthy especially Spanish participants, seem to pay more attention to the price of seafood while the degree of scientific knowledge appears not to affect the overall purchasing decision. In the case of the Spanish consumers, this can be explained by the long fishing tradition in the country, and as Fernández-Polanco and Luna (2012) showed, hence price is the only favourable factor towards purchasing aquaculture products over wild capture-fisheries products. Thus, price perception as purchasing decision factor emerges as the main incentive in regard to fish consumption between countries and user groups with different consumption profiles (Ingram, 2017; SAPEA, 2017). Especially when money is involved, it is a widespread phenomenon across all participants to favour the price above all other criteria. This points to the central problem of willingness-to-pay studies in seafood consumption studies (Chang and Nguyen, 2018; Grunert et al., 2009; Zander and Feucht, 2018). More often than not, studies revealed that individuals state that they ignore the price of a high-quality product (see e.g., van Osch et al., 2017; Xuan and Sandorf, 2020; Yip et al., 2017). However, these statements do not match actual shopping figures from supermarkets (in Germany and elsewhere), which show that consumers, to a considerable extent, tend to buy the cheapest product on the shelf without considering its origin or quality (Eurobarometer, 2018; NSC, 2019). In conclusion, in our survey we could neither see country nor scientific knowledge related differences concerning the price as purchasing criterion for seafood products.

#### 3.3.2 Origin preferences

More than 70% of all respondents to our surveys have begun to consider the origin of the fish. In the semi-structured interviews, particularly with participants with a higher level of scientific knowledge, it was stated that these were more selective with respect to the origin of the seafood product purchased. The interviews further revealed that these consumer groups tend to pay more attention to the regionality of the product, which plays an essential role in the concern of supporting sustainability. This mirrors the findings by Guillen et al. (2019), who demonstrated that competing for resources is a rising issue in finfish production for consumption, and more consumers are willing to emphasize their awareness of sustainability by focusing on the origin of the product. Aquaculture is known to provide a more efficient production system than wild-capture finfish, as well as to be far more sustainable, primarily when a circular economy approach is applied (Regueiro et al., 2021). Our study showed that it can be assumed that more focus on the regionality dimension of aquaculture could improve the demand for aquaculture products, especially if these are produced as part of a circular economy set up. However, consumer concerns and assumptions about regionality and sustainability need to be considered in the promotion and/or production of cultivated fish.

### 3.4 Rethinking the pathways to sustainable seafood consumption in Europe

Today's societies have evolved into multi-layered, highly fragmented and diverse entities with a wide range of interests, views, knowledge structures, perspectives, norms and values (Jacobs et al., 2015). These are also mirrored in the decision-making process of seafood purchasing. This makes addressing our last research question challenging, that is how we can shift consumers behaviour towards a more sustainable purchasing behaviour. To this end, the social dimensions of consumer acceptance and their related individual purchasing decisions must be tackled.

Our study reinforces findings from previous research (such as Asche and Bronnmann, 2017; Fernández-Polanco and Luna, 2012; Karnad et al., 2021), which showed that the degree of scientific knowledge about seafood products does not automatically influence purchasing behaviour. For instance, Fernández-Polanco and Luna (2012) identified the socio-demographic background, product promotion, and price as the three most crucial seafood purchase criteria. Our results are consistent with their findings in that regard that price appears to be the most important purchase criterion across all countries investigated.

If we conclude that increased knowledge does not necessarily lead to more aquaculture products being purchased, the question remains as to what would support a change towards more sustainability-oriented purchasing behaviour. The results of the semi-structured interviews conducted in this study revealed that individuals with a high level of scientific knowledge about seafood products are highly interested in the sustainability of the fish they consume. However, this increased awareness does not necessarily translate into direct behaviour patterns in the supermarket when these individuals purchase fish for personal consumption (Lawley et al., 2019). Furthermore, the current multitude of different certification systems certified product labelling often does not clearly identify sustainability aspects hence causing confusion among consumers. Indeed, the wide range of certification labels leads to misunderstandings, and people eventually resign themselves to trying harder to understand the label (Alfnes et al., 2018). Interestingly, the results of our semi-structured interviews indicated that the more people know about a respective certification system, the less they stated to trust it. However, people who are less scientifically knowledgeable about seafood production stated to trust some labels and indicated to become more confused when shopping at the supermarket. One participant at the KTE 1 described feeling that there is an “ecolabel jungle”, with more and more ecolabels popping up almost weekly, promising to be more sustainable and environmentally friendly than the others. In this regard our semi-structured interviews exposed a major problem in purchasing in regard to transparency and traceability of the product. These issues tend to be overlooked and overseen by the producers of aquaculture products but play an important role for the individual purchasing behaviour disregarding the country-specific context nor the degree of available scientific knowledge. Thus, the hypothesis that more scientific knowledge leads to more sophisticated purchasing criteria may be appealing from the onset, but limitations emerge when applying this hypothesis to real-world contexts. The issues of food labelling and confusion in respect to how products are certified reduce the actual intended outcome towards more sustainability‑lead purchasing behaviour are a case in point (Ihemezie et al., 2018). The question of how to overcome this problem remains, and more studies are needed that focus on a different way of how to nudge and change purchasing behaviour towards a more sustainable product choice. These must be tailored to country-specific settings and to their respecting processing preferences.

The results of this study hence call into question current strategies for promoting sustainable aquaculture products. The more different promotional options are offered, the more consumers appear to become confused and eventually resign from the effort to consider sustainability aspects of the product in their purchasing decisions (Ihemezie et al., 2018; Maesano et al., 2019). To overcome this barrier, one option could be to develop a coherent national (or even international) strategy for seafood marketing and more specifically, for sustainable aquaculture. Apparently, as indicated by our results, it is essential to develop a non-confusing option that helps consumers understand sustainability without further confusion. A potential route is the “Nutri Score” developed by the Federal Ministry of Food and Agriculture in Germany. On a range between 1 and 5 the score indicates the nutritional value for each product (Schlögl, 2020). Labelling the product with the score is voluntary, and manufacturers can decide whether or not to use it. Despite that this may not be the perfect solution and is yet contested, it provides consumers more detailed information about the food and nutritional values at first glance in a straightforward manner. A similar labelling system for seafood products, where the sustainability of the product as well as the production circumstances are easier to capture could possibly be a more effective support towards sustainability-led purchasing decisions than 100 different ecolabels. This is mirrored by Maesano et al. (2019) who provided evidence in their literature review that people generally have a higher willingness-to-pay for a product even when they do not fully understand the label but assume that it is a sustainably produced product.

In addition, the current focus on circular economy approaches is another avenue to promote more sustainable and transparent products. Under this umbrella, the aquaculture sector can be assumed to be more economically competitive, if it allows better circular options of production. In this way, wild fish stocks can be conserved while ecosystem services are improved, leading to better overall consumer perception (Ruiz-Salmón et al., 2020).

### 3.5 Limitations of the study methodology

This study was conducted as part of the GAIN project and serves as the first entry point to understand the role of scientific knowledge and related consumer purchasing behaviour. To that end, it focuses exclusively on the level of scientific knowledge on seafood products, particularly on sustainable aquaculture products, and how specific levels of available scientific knowledge influences consumers purchasing decisions.

However, all social science surveys addressing personal perspectives inherit a factor of error, especially when enquiring into rather normative reasoning of individual understanding of sustainability that can be mostly captured only in a qualitative manner. The distorted perception of sustainability on the individual level as well as the rather coarse categorization of the scientific knowledge levels per individual participant leads to limitations in the scientific rigor of the findings, next to the fact that many participants are not fully honest in their statements made (Bursztyn et al., 2019; Solgaard and Yang, 2011; Zander and Feucht, 2018).

That said, our experience showed that the information captured on the poster survey did not always reflect the perspectives voiced in the subsequent semi-structured interviews. In this regard, the effect of peer pressure (Shu, 2018; Vinayak and Arora, 2018) is particularly noteworthy and surfaced strongly the more people gathered together in small groups to view and complete the poster survey at the same time. A good example of the emergence of this type of peer pressure was in closed events, such as KTE 3, where participants knew each other well and were asked their opinion on the role of price in their purchasing decision. On these occasions, roughly 81% stated to ignore price and focus on other (mainly sustainability-led) buying criteria. This can be explained by the influence of peer group pressure, as Vinayak and Arora (2018) have shown. It can be expected that participants at these particular KTEs do pay attention to price but were embarrassed to indicate it openly among their peers. For instance, the participants at KTE 3 all had intermediate scientific knowledge about seafood and were well aware of the adverse effects of looking only at the price as purchasing decision.

Another limitation is that consumers often misjudge how much they know and do not know about a given subject (Krešić et al., 2020). In our semi-structured interviews, participants often initially claimed to know about the sustainability of seafood products, especially on aquaculture products. However, in the course of the interviews, more often than not the claimed knowledge surfaced to be erroneous when inquiring in more detail about specific sustainability attributes. This overestimation of own (scientific) knowledge often leads to contestation of new knowledge rooted in scientific evidence. Many of the participants interviewed were reluctant to learn about new evidence about sustainable aquaculture, thus showing signs of being conditioned to be highly sceptical of the information they receive resulting in preferring to remain in well-trained purchasing patterns. This somewhat is in contrast to the yet prevailing view that finding scientific facts informs actions. Investigating this discrepancy and the very implications for seafood consumption appears to be a crucial under-researched issue. Further studies need to show the extent to which this knowledge gap impacts consumption.

### 3.6 Future recommendations

Looking critically into the future, we see that Generation Z, born in the digital age between 1995 and the early 2000s, is to date the largest consumer group in the world (Su et al., 2019; Zuo et al., 2022). This generation places a high emphasis on environmentally-friendly foods, sustainability and animal welfare (Su et al., 2019; Zuo et al., 2022). Therefore, the generational differences in purchasing behaviour need to be considered in future campaigns (Kamenidou et al., 2020). That being said, do most campaigns in the European union do neither focus on generational nor country-specific differences. This strategy needs to be challenged if more awareness and promotion with regard to the sustainability of purchasing decisions of seafood products, specifically aquaculture products, is the goal. A first approach could be to show (especially for Generations Z and Y) the “new” production system, the so-called circular economy approaches. Studies have shown that the younger generation is especially interested in sustainability (carbon footprint) and upcycling of food to avoid food crises and waste (Kymäläinen et al., 2021; Zhang et al., 2021).

Another aspect for future studies is the application of interactive posters surveys, which is method still very underutilized in the field of aquaculture and consumer perceptions research. This study demonstrated an initial approach to incorporating interactive poster surveys at conferences to gain valuable insights into attendee perspectives. We would recommend using these interactive posters in awareness campaigns to better understand the rather intangible reasoning of consumers and their purchasing decisions, vis á vis to spark their interest in aquaculture products. A study by Michalsky et al. (2018) examined an interactive approach to changing attitudes towards people living in poverty. The study used social media and interactive installations to “put the user in the shoes of someone living in poverty.” The interactive engagement of participants generated an emotional response and led to greater understanding. It also fostered a relationship between people experiencing poverty and the study participants. Using this study as a model, we would make a similar recommendation for the future. For instance, one approach could be to show seafood buyers the different living conditions of a fish in the wild and a fish in aquaculture, focussing on the health benefits of farmed animals (fewer toxins, fewer sick animals), sustainability (no bycatch, usually poor working conditions in fisheries), and reuse of resources (circular economy). Such an approach may potentially foster a similar emotional response from participants, leading to a change in purchasing behaviour.

## 4 Conclusion

Preferences are highly related to a rather intangible set of individual-cultural context-dependent drivers that somewhat contradict the affirmative role of scientific knowledge in a healthy information society. To this end, we are aware that this study provides only a first impression on this subject and further and more detailed research is needed to prove the here stated results. In conclusion, the rapid changes in consumer demographic, social, and economic structures and production patterns call for new research on translating information and awareness into practice. Our study demonstrated that consumers' beliefs, norms and values all effect personal perceptions and consumption of aquaculture products. These often appear to be more important than other categories such as animal welfare, price, or origin. Perceptions and purchasing habits are dynamic and vary from culture to culture. A lack of clear and accessible information can generally be considered the main barrier to the social acceptance of aquaculture products in Europe. Potential country specific differences in species composition preference could be identified. In contrast, results on the role of scientific knowledge were rather blurred. Some participants in this study exhibited a high level of scientific knowledge about aquaculture products while still choosing the less sustainable product option due to financial reasons. This indicates that price appears to be the most important purchasing criteria. Next, we found that younger people with less scientific knowledge about seafood were more likely to adopt a vegan/vegetarian lifestyle.

We conclude that food and, more specifically, country-specific food culture plays an important societal role. By understanding consumer preferences in different EU countries and using diverse scientific evidence, opportunities to transform current marine food systems in the EU may emerge. However, more data are needed to provide further information on the relationship between scientific knowledge, food culture and respective purchasing behaviour. Our findings raise the question whether trying to educate people about more sustainable purchasing criteria is really the key to more sustainable purchasing or are we, based on wrong assumptions, applying wrong approaches to foster more sustainability-led purchasing decisions.

## CRediT authorship contribution statement

**J. Petereit:** Conceptualization, Methodology, Validation, Formal analysis, Investigation, Resources, Data curation, Writing – original draft, Visualization. **C. Hoerterer:** Conceptualization, Methodology, Validation, Investigation, Writing – review & editing. **G. Krause:** Conceptualization, Methodology, Validation, Investigation, Resources, Writing – review & editing, Supervision, Project administration, Funding acquisition.

## Declaration of Competing Interest

The authors declare that they have no known competing financial interests or personal relationships that could have appeared to influence the work reported in this paper.
